# The realization of the dipole (*γ, γ*) method and its application to determine the absolute optical oscillator strengths of helium

**DOI:** 10.1038/srep18350

**Published:** 2015-12-17

**Authors:** Long-Quan Xu, Ya-Wei Liu, Xu Kang, Dong-Dong Ni, Ke Yang, Nozomu Hiraoka, Ku-Ding Tsuei, Lin-Fan Zhu

**Affiliations:** 1 Hefei National Laboratory for Physical Sciences at Microscale and Department of Modern Physics, University of Science and Technology of China, Hefei, Anhui 230026, People’s Republic of China; 2 Synergetic Innovation Center of Quantum Information and Quantum Physics, University of Science and Technology of China, Hefei, Anhui 230026, People’s Republic of China; 3 Shanghai Institute of Applied Physics, Chinese Academy of Sciences, Shanghai 201204, People’s Republic of China; 4 National Synchrotron Radiation Research Center, Hsinchu 30076, Taiwan, Republic of China

## Abstract

The dipole (*γ, γ*) method, which is the inelastic x-ray scattering operated at a negligibly small momentum transfer, is proposed and realized to determine the absolute optical oscillator strengths of the vanlence-shell excitations of atoms and molecules. Compared with the conventionally used photoabsorption method, this new method is free from the line saturation effect, which can seriously limit the accuracies of the measured photoabsorption cross sections for discrete transitions with narrow natural linewidths. Furthermore, the Bethe-Born conversion factor of the dipole (*γ, γ*) method varies much more slowly with the excitation energy than does that of the dipole (e, e) method. Absolute optical oscillator strengths for the excitations of 1s^2^ → 1 s*n*p(*n* = 3 − 7) of atomic helium have been determined using the high-resolution dipole (*γ, γ*) method, and the excellent agreement of the present measurements with both those measured by the dipole (e, e) method and the previous theoretical calculations indicates that the dipole (*γ, γ*) method is a powerful tool to measure the absolute optical oscillator strengths of the valence-shell excitations of atoms and molecules.

The absolute optical oscillator strength (OOS), or the equivalent quantity of photoabsorption cross section, of an atom or molecule represents the transition probability between two quantum states and is essential in describing and understanding the physical processes involving the photon absorption and emission, which widely exist in plasma, interstellar space, planetary atmosphere, and energy deposition. Therefore, absolute OOSs of atoms and molecules with high accuracy are crucial for the development of the related subjects, and have attracted continuous attention from both theorists and experimentalists. Meanwhile, precise experimental OOSs can be used to test the theoretical models and calculational codes rigorously. Thus far, substantial experimental efforts have been made to improve the acurracy and precision of the OOSs. However, discrepancies still exist, even for some simple atoms and diatomic molecules^1^,^2^,^3^,^4^,^5^,^6^,^7^,^8^,^9^,^10^. It is hence desirable to develop a new experimental method to measure the absolute OOSs of atoms and molecules, especially for valence-shell excitations; this is the focus of this paper.

Different types of experimental techniques, including the photoabsorption method^1^,^2^,^3^,^4^, electron energy loss spectroscopy (EELS)^5^,^6^,^11^, dipole (e, e) method^7^,^8^,^9^,^12^,^13^,^14^,^15^, lifetime method^16^,^17^, and selfabsorption^18^,^19^,^20^, have been used to measure the absolute OOSs of atoms and molecules. Among them, the photoabsorption method and the dipole (e, e) method are most commonly used. In principle, the photoabsorption method based on the Beer-Lambert law is the simplest and most straightforward technique to measure the absolute OOSs of atoms and molecules. It is well known that the photoabsorption method can provide the accurate absolute OOS density for the continuum region and the OOSs of discrete states with wider natural widths such as the dissociative states of molecules. However, most discrete excitations of atoms and molecules have very narrow natural line widths (much smaller than the finite energy resolution of the spectrometer), and it is in general difficult to determine their OOSs accurately when using the photoabsorption method because of the line saturation effect^14^,^21^. As a result, the experimental OOS for a definite excitation measured by the photoabsorption method is always smaller than the real value, and the line saturation is even more serious for the stronger and narrower transition. The line saturation effect of the photoabsorption method has been discussed in detail by Chan *et al.*^14^, and it can be eliminated by extrapolating the measurements at different target pressures to zero pressure, or improving the experimental energy resolution substantially^22^. Although the photoabsorption method can yield accurate OOSs for the discrete excitations of atoms and molecules after rigorous corrections, the line saturation effect should be corrected carefully because it is not invariable for different transitions.

The dipole (e, e) method is another commonly used technique to measure OOSs of atoms and molecules. It can trace its history to van der Wiel’s works in the 1970’s^23^,^24^,^25^, while the full establishment of this method should be attributed to Brion and his coworkers^14^,^26^. In the dipole (e, e) method, the high energy electron impact (~several keV) is used and operated at *q*^2^ ≈ 0 (i.e., at a small scattering angle) to simulate the photoabsorption process. The dipole (e, e) method has the advantage of being free from the line-saturation effect in determining the optical oscillator strength because of the nonresonant nature of the electron-impact excitation process. Being low cost, the electron energy loss spectrometer was once called the “poor-man’s synchrotron”^27^, and has provided a large body of absolute OOSs for various atoms and molecules. In the 1970’s, the dipole (e, e) method was limited by its low energy resolution and the normalization process, and the latter was dependent on the absolute OOS density in the continuum region measured by the photoabsorption method or calculated by theorists. In the 1990’s, Brion and his collaborators^14^,^26^ used the Thomas-Reiche-Kuhn (TRK) sum rule to absolutize the measured spectrum and an independent experimental technique, i.e., the dipole (e, e) method, was established. At the same time, Brion and his coworkers developed the high resolution (approximately 48meV) dipole (e, e) method^14^,^26^, and many absolute OOSs for the vallence-shell excitations of atoms and molecules have been reported by their group^7^,^8^,^9^,^10^,^14^,^15^. It is worth noting that the absolute OOSs measured by the dipole (e, e) method provide a cross-check to the ones determined by other experimental methods and promote this field greatly.

In addition to the above-mentioned photoabsorption and dipole (e, e) methods operated at an optical limit, the OOS can be determined by extrapolating the measured generalized oscillator strength (GOS) in *q*-space to *q*^2^ ≈ 0^5^,^6^,^11^. For the dilute gas target in atomic and molecular physics, the GOS is measured by the EELS method due to its advantage of having large cross sections, but this method suffers from the tedious procedures of measurement and extrapolating for each transition. With regard to the dense target in condensed matter physics, the dynamic structure factor *S*(*q, ω*)^28^,^29^ in *q*-space can be measured by the inelastic x-ray scattering technique because its small cross section can be complemented by the large target density. From the obtained *S*(*q, ω*), the optical oscillator strength distribution, the optical energy-loss function (OLF), the complex dielectric function, the complex index of refraction, and the reflectance can be derived^30^,^31^,^32^,^33^. The electron inelastic mean free path (IMFP)^34^,^35^, which is a parameter of fundamental importance to a range of fields including electron microscopy, Auger electron spectroscopy, x-ray photoelelctron spectroscopy and photoelectron diffraction, can be obtained from the OLF determined at *q*^2^ ≈ 0 by the inelastic x-ray scattering (IXS)^36^. Furthermore, the OLF measured by the IXS provides a cross-check to the theoretical calculations and the experimental ones determined by other techniques such as the overlayer technique, fluorescent measurement, and the extraction from XAFS and absorption^36^,^37^. The dipole (e, e) method and those mentioned above provide some inspiration for this work.

In this paper, we propose and realize the high resolution dipole (*γ, γ*) method to measure the absolute OOSs of valence-shell discrete excitations for gaseous atoms and molecules by utilizing the inelastic x-ray scattering technique at *q*^2^ ≈ 0. With the independent nomalization procedure, the absolute OOS data can be determined accurately using the present dipole (*γ, γ*) method, which is discussed in detail below. Then we measure the absolute OOSs of valence-shell excitations of atomic helium with the dipole (*γ, γ*) method and compare them with the previous experimental and theoretical results.

## Theoretical Background

According to quantum electrodynamics, the diferential cross section (DCS) of the IXS for gaseous atoms and molecules can be written as^28^,^38^,^39^,^40^ (the atomic unit is used throughout this paper):


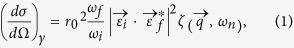


where the subscript *γ* represents the photon scattering process. Equation (1) is valid for inelastic x-ray scattering with polarized incident photons. 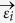 and 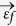 are the polarization vectors of incident and scattered photons, respectively. *r*_0_ is the classical electron radius. *ω*_*i*_ and *ω*_*f*_ are the energies of incident and scattered photons, respectively. 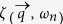 is the so-called squared form factor, which is the square of the transition matrix element^41^,^42^,^43^,^44^:


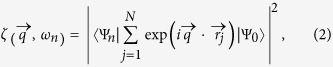


where 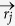 is the position vector of the *j*th electron in the target. Ψ_0_ and Ψ_*n*_ represent the initial and final wave functions of the target, respectively. 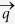 is the vector of momentum transfer and *N* is the number of electrons of the target. Z-axis can be chosen for any target, it is for atoms that it leads to the greatest simplifications due to the spherical symmetry. Thus, equation (2) is written as:


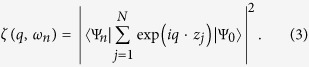


If the scatterer is a gaseous molecule, *ζ*(*q, ω*_*n*_) should be averaged over the orientations of the molecular axis. For convenience, we discuss the atom case in detail, and the obtained consequence is also valid for molecules.

When the momentum transfer is very small, the transition matrix element in equation (3) can be expanded as:


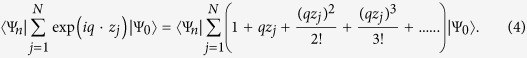


The first term on the right-hand side in equation (4) is zero due to the orthogonality of the initial and final wave functions for an excitation process. Considering that *q* is very small and neglecting the small quantities of the second and higher orders, the transition matrix element obtained above is:


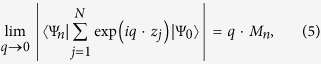


where


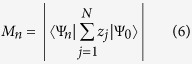


is the dipole-matrix element. Therefore, at small momentum transfer, i.e., at small scattering angle, the inelastic x-ray scattering technique can be used to simulate the photoabsorption process and we call it the dipole (*γ, γ*) method. Accordingly, we can use the inelastic x-ray scattering technique to measure the absolute OOSs for atoms or molecules.

Combining equations (1) and (5) and the definition of the OOS, the important relationship between the OOS and DCS of the present proposed dipole (*γ, γ*) method is


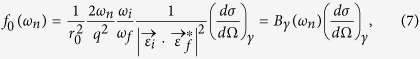


where


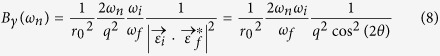


is the Bethe-Born factor that can be considered a kinematic factor. The Bethe-Born factor connects the inelastic x-ray scattering spectrum and the absolute optical oscillator strength of the target at negligible momentum transfer. As a result, the momentum transfer should be considerably small so we can convert the DCS of the inelastic x-ray scattering into the absolute optical oscillator strength by utilizing equation (7) exactly. In fact, at *q*^2^ ≤ 10^−2^ a.u. equation (7) is satisfied to better than 1% accuracy as noted by Chan *et al.*^14^, and the contribution of the high-order terms in equation (4) to the OOS is much smaller than the experimental uncertainties, which is shown in detail below. Similarly, at negligible momentum transfer, Brion and his colleagues successfully applied the electron scattering technique that is usually called the dipole(e, e) method to measure the absolute OOSs of valence-shell excitations for atoms and molecules^26^.

## Experimental Method

**Figure 1:**
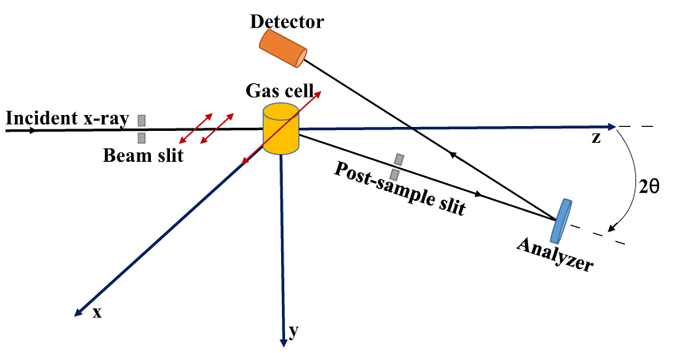
Schematic of the experimental setup of the inelastic x-ray scattering of gases. The incident beam is monochromatized by a Si(333) monochromator with a resolution of 50 meV and the polarization direction of the incident photon is along the x axis in the horizontal scattering plane. The target gas is sealed in the gas cell with kapton windows through which the incident and scattered photons pass in and out. After collisions, the scattered photons will pass through a post-sample slit before being collected by the crystal analyzer, which can reduce the background efficiently. The scattered photons were collected and dispersed by a spherical bent crystal analyzer [Si(555) with a 2m radius of curvature] and detected by an AMPTEK XR100CR detector above the gas cell. In sum, the total energy resolution in the experiment is 70 meV.

The present IXS measurement of helium was carried out at the Taiwan beamline BL12XU of SPring-8, and the schematic of the experimental setup is shown in Fig. 1. The same setup has been used to study the squared form factors of valence shell excitations of atoms and molecules in our previous works^44^,^45^,^46^,^47^. In the present experiment, the analyzer energy for the scattered photon was fixed at 9889.37 eV, whereas the incident photon energy varied, from which the energy loss can be deduced easily. Considering the finite angular resolution, the average *q*^2^ was estimated to be 0.010 a.u. at the scattering angle of 2°. The intensity of the incident x-ray is monitored through a silicon p-i-n diode in front of the gas cell and its fluctuation can be corrected accordingly. In the actual measurement, we record the counts of scattered photons for a discrete excitation, whose differential cross section can be determined by^44^:


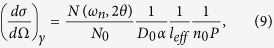


where 2*θ* is the scattering angle. *N*(*ω*_*n*_, 2*θ*) and *N*_0_ represent the counts of the scattered photons and the intensity of incident photons, respectively, and the former is obtained by fitting the raw experimental spectrum. *D*_0_ is a factor that is determined by the detection efficiencies of the ionization chamber and the detector of the scattered photon, and it can be taken as a constant because the measured energy loss region is much smaller than the incident photon energy. The transmissivity *α* is determined by the sample species and its pressure, and it can be measured accurately both with and without sample gas in the gas cell by an ionization chamber after collision. *l*_*eff*_, *n*_0_, and *P* are the collision length, density of the target at 1 atm, and pressure of the target in units of atm, respectively. Due to the finite solid angle of the analyzer, the experimental differential cross section is the result of convolution of the real differential cross section and the angular resolution function of the spectrometer. Thus, the angular resolution should be considered for the Bethe-Born factor accordingly, and we call it the Bethe-Born conversion factor:


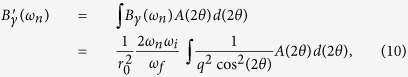


where *A*(2*θ*) is the angular resolution function of the spectrometer, which can be determined accurately by simulating the actual arrangement of the light path in consideration of the rectilinear propagation of light.

Inserting formula (9) into formula (7) and replacing *B*_*γ*_(*ω*_*n*_) with 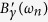, the relative optical oscillator strength is given as:


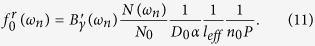


To obtain the absolute OOS of the atom and molecule, a simple normalization method is used:


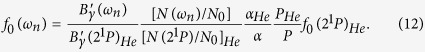


Herein, the OOS of 2^1^*P* transition of helium as a calibration standard is due to helium being the simplest multielectron system, and the calculated OOS of the 2^1^*P* transition has reached a very high accuracy of approximately 1 × 10^−6^ 48. The spectrum of the transition *n* to be studied should be measured at the same experimental condition as that of the 2^1^*P* transition of helium. Therefore, *l*_*eff*_ and *D*_0_ in equations (9) and (11) can be considered constants, and have been eliminated in equation (12).

**Figure 2:**
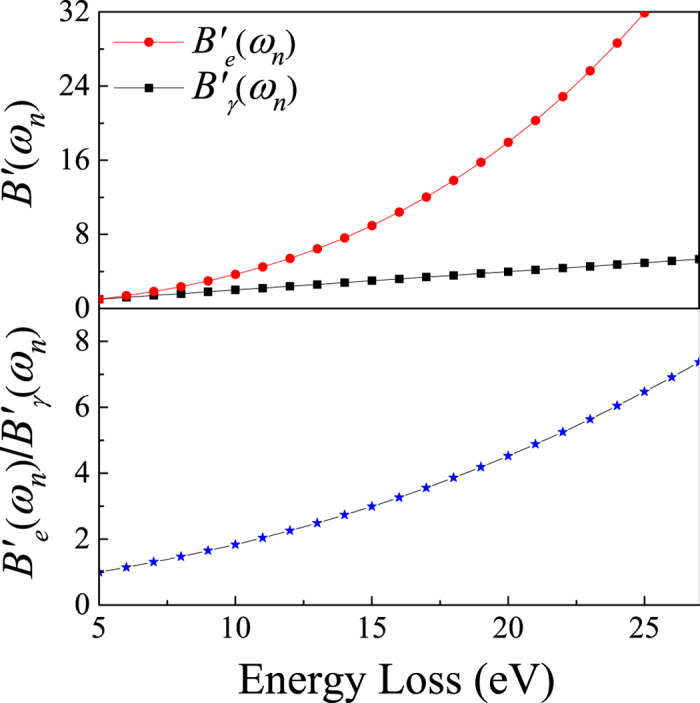
The Bethe-Born conversion factors of the dipole (*γ, γ*) method 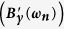 determined by simulating the light path of the present experimental setup, and the high-resolution dipole (e, e) method 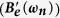 produced according to the typical parameters in Chan *et al*.^14^. The vertical values are in arbitrary unit and have been normalized to a point at the energy loss of 5 eV.

According to equation (12), the experimental errors of absolute OOSs come from the statistical counts and the Bethe-Born conversion factors of transitions *n* and 2^1^*P*, and from the transmissivities and the pressures of the target and helium. The raw spectrum of helium was fitted using the PeakFit software program, from which the statistical errors caused by the least-squares fitting can be obtained. For different transitions (2^1^*P* − 7^1^*P*), the statistical errors are estimated to be approximately 0.7–4%. The measured accuracy of the transmissivity is better than 0.1%. The pressures were measured by a commercial digital pressure manometer with a declared accuracy of better than 0.55%. For this work, only helium gas is used, so the transmissivity *α* and the gas pressure are constants and provide no contribution to experimental errors. For the present dipole (*γ, γ*) method, the Bethe-Born conversion factors have been accurately determined by simulating the light path directly according to equation (10) and shown in Fig. 2. It can be found that 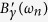 varies slowly with the excitation energy and this rising tendency is due to the excitation energy which is accurately known. In the present dipole (*γ, γ*) method, the high precision Bethe-Born conversion factor, whose accuracy is believed to be better than 0.2%, will not introduce noticeable errors. Similar to the dipole (*γ, γ*) method, the Bethe-Born conversion factor is also used in the calibration process of the dipole (e, e) method (marked as 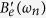)^14^. Due to the complexity of the electron optics, it is difficult to trace the electron path in the high-resolution dipole (e, e) method. Therefore, 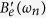 was determined by fitting the high-resolution dipole (e, e) spectrum in the ionization continuum region divided by the OOS density in the same region measured by the low-resolution dipole (e, e) spectrometer^14^. 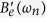 at low *ω*_*n*_ is derived by extrapolating the Bethe-Born conversion factors from above 24.6 eV to *ω*_*n*_. The relative 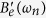 calculated with the typical parameters given in Chan *et al.*^14^ is shown in Fig. 2, and it is obvious that 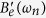 changes rapidly with *ω*_*n*_. Considering that the valence-shell excitation energies of atoms and molecules are commonly smaller than 24.6 eV (such as 12.5 eV for *b*^1^Π_*u*_ (*ν*′ = 0) of nitrogen and 6.8 eV for the Schumann-Runge continuum of oxygen), the rapidly varied 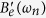 may bring considerable uncertainties. Furthermore, these potential uncertainties are difficult to evaluate and not included in the results of the dipole (e, e) measurement.

## Results and Discussion

**Figure 3:**
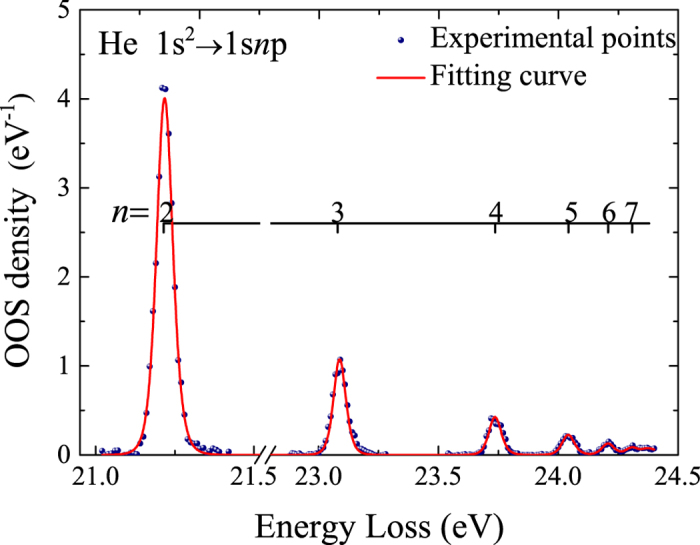
The IXS spectrum of helium with the valence-shell excitations assigned. Solid blue circles: the present experimental data; red line: fitted results. The vertical axis has been converted into absolute OOS density.

**Figure 4:**
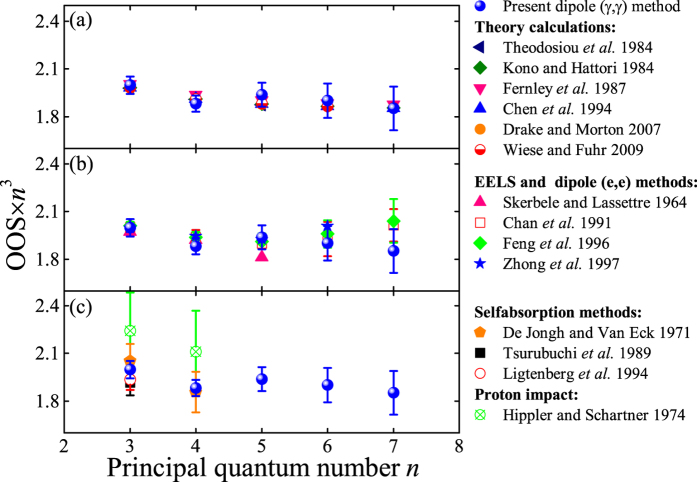
The comparison of the present results with: (**a**) the different calculations; (**b**) the experimental results by the EELS and dipole (e, e) methods; (**c**) the experimental results measured by the selfabsorption methods and proton impact method.

In the present work, the 1*s*^21^*S*_0_ → 1*snp*^1^*P*_1_ series (*n* = 3–7) of helium were measured and the obtained spectrum is shown in Fig. 3 along with the excited states assigned. With the energy resolution of 70 meV, the excitations are resolved clearly. The absolute optical oscillator strengths of the 1*s*^21^*S*_0_ → 1*snp*^1^*P*_1_ series (*n* = 3–7) for helium determined by this work are listed in Table 1 and shown in Fig. 4.

It is clear that in Fig. 4(a), most of the latest calculations, i.e., Coulomb approximation with a realistic central field by Theodosiou *et al.*^49^, the double Hylleraas-type basis functions by Kono and Hattori^50^, B-spline basis functions by Chen *et al.*^51^,^52^, high-precision variational calculations considering singlet-triplet mixing and spin-orbit coupling by Drake and Morton^53^, and the extension of Drake and Morton^53^ using higher precision experimental wavelengths listed in the NIST ASD by Wiese and Fuhr^54^, give nearly identical results, whereas the results of close coupling by Fernly *et al.*^55^ are slightly higher. In sum, the present OOSs by the dipole (*γ, γ*) method agree excellently with these sophisticated calculations. Nevertheless, the present results for excitations to 3^1^*P*, 5^1^*P* and 6^1^*P* states are slightly larger than the calculated values, whereas those of 4^1^*P* are slightly smaller, as shown in Fig. 4(a).

Within the errors, all of the present results measured by the high-resolution dipole (*γ, γ*) method are in excellent agreement with those measured by Chan *et al.*^14^, Feng *et al.*^13^ and Zhong *et al.*^12^ with the dipole (e, e) method and with the results obtained by Skerbele and Lassettre^56^. Generally speaking, the results of the dipole (e, e) method are slightly larger than the above mentioned calculated ones, especially for the values of 6^1^*P* and 7^1^*P* transitions, whereas our dipole (*γ, γ*) method results are in better agreement with these calculations. Most results of the selfabsorption methods^18^,^19^,^20^, restricted to low *n* values (see Fig. 4(c)), are in agreement with both the calculations and the present results. On the other hand, the earlier measurement performed by Hipper and Schartner^57^ using the proton impact method disagrees with the others.

## Conclusion

In this work, the dipole (*γ, γ*) method is proposed and realized to measure the absolute optical oscillator strengths of the valance-shell excitations of atoms and molecules. The measured absolute OOSs of 1*s*^21^*S*_0_ → 1*snp*^1^*P*_1_ series (*n* = 3–7) for helium are all in excellent agreement with the sophisticated calculations and most experimental measurements in literatures. The present results provide a rigorous test of the theoretical methods and confirm the validity of the high resolution dipole (*γ, γ*) method. Because the dipole (*γ, γ*) method is not subject to the line-saturation effect and its Bethe-Born conversion factor varies much slowly with the excitation energy compared with that of electron-impact method, the OOS determined by the dipole (*γ, γ*) method can serve as benchmark data and provide a cross-check to those measured by the optical method and the dipole (e, e) method, especially for the transitions with very low excitation energies. The present work shows that the dipole (*γ, γ*) method based on the inelastic x-ray scattering technique at the third generation synchrotron radiation is a powerful tool to determine the absolute optical oscillator strengths of valence-shell excitations of other atoms and molecules.

## Additional Information

**How to cite this article**: Xu, L.-Q. *et al.* The realization of the dipole (γ, γ) method and its application to determine the absolute optical oscillator strengths of helium. *Sci. Rep.* **5**, 18350; doi: 10.1038/srep18350 (2015).

## Figures and Tables

**Table 1:** Theoretical and experimental determinations of the absolute dipole oscillator strengths for the (1*s* ^21^ *S*_0_ → 1*snp* ^1^ *P*_1_) transitions in helium.

1^1^*S* →	3^1^*P*	4^1^*P*	5^1^*P*	6^1^*P*	7^1^*P*
**Dipole(*****γ**, **γ*****) method:** (this work)	0.0740 (0.0020)	0.0294 (0.0008)	0.0155 (0.0006)	0.0088 (0.0005)	0.0054 (0.0004)
**Theoretical calculations:**					
Wiese and Fuhr 2009^54^	0.07346	0.02987	0.01505	0.00863	0.00541
Drake and Morton 2007^53^	0.07344	0.02986	0.01504	0.00863	0.00541
Chen *et al.* 1994^51^,^52^	0.07342	0.02986	0.01503	0.00863	0.00540
Fernley *et al.* 1987^55^	0.07434	0.03028	0.01524	0.00873	0.00547
Kono and Hattori 1984^50^	0.07344	0.02987	0.01504	0.00863	0.00541
Theodosiou *et al.* 1984^49^	0.07334	0.02981	0.01500		
**Electron impact methods**:					
Zhong *et al.* 1997^12^	0.0739	0.0304	0.0154	0.0093	
Feng *et al.* 1996^13^	0.0745 (0.0009)	0.0303 (0.0005)	0.0153 (0.0004)	0.00907 (0.0004)	0.00595 (0.0004)
Chan *et al.* 1991^14^	0.0741 (0.0007)	0.0303 (0.0007)	0.0152 (0.0003)	0.00892 (0.0005)	0.00587 (0.0003)
Skerbele and Lassettre 1964^56^	0.073	0.030	0.0145		
**Selfabsorption:**					
Ligtenberg *et al.* 1994^18^	0.0717 (0.0024)				
Tsurubuchi *et al.* 1989^19^	0.071 (0.003)				
De Jongh and Van Eck 1971^20^	0.076 (0.004)	0.029 (0.002)			
**Proton impact method:**					
Hippler and Schartner 1974^57^	0.083 (0.009)	0.0330 (0.004)			

Estimated uncertainties in experimental measurements are shown in parentheses.
